# Female expertise in public discourses: Visibility of female compared to male scientific experts in German media coverage of eight science-related issues

**DOI:** 10.1177/09636625251363937

**Published:** 2025-09-07

**Authors:** Melanie Leidecker-Sandmann, Nikolai Promies, Markus Lehmkuhl

**Affiliations:** Karlsruhe Institute of Technology, Germany

**Keywords:** content analysis, diversity, gender, media coverage, risk communication, science communication

## Abstract

A fair (public) representation of women is one of the most discussed questions of our time. The way in which media coverage (re)produces genders may affect individual and collective thinking and the perceptions of women in society. We analyse the representation of female scientists in German news media coverage of eight science-related risk issues and compare male and female experts regarding their relative scientific reputation, the number of references and the content of their statements. Our findings show that female scientific experts are less visible in German media coverage than their male colleagues and that they are underrepresented compared to the respective proportions in the relevant research areas. At the same time, our data relativize the extent of the gender visibility gap – after controlling for hierarchical position and scientific reputation, the differences become rather small. We find no evidence of discrimination against female scientific experts through journalistic selection routines.

## 1. Introduction, research interest and relevance

The fair representation of women, for example in media coverage, is referred to as one of the most discussed questions of our time (Trepte and Loths, 2020) and as a challenge for journalism. Media play a pivotal role in shaping public perception, particularly through the lens of constructivist theory, which emphasizes the socially constructed nature of reality. From this perspective, (mass) media do not simply reflect reality but actively construct it by selecting which voices are heard and which narratives are emphasized (Tuchman, 1978). Cultivation theory suggests that media coverage may affect audiences’ perceptions of social reality (Gerbner and Gross, 1976), including notions of who is meaningful or relevant for society and who is not (Fryberg and Townsend, 2008). In sum, the way in which media coverage (re)produces gender affects individual and collective thinking and the assessment and perceptions of women in society (GMMP, 2020; Magin and Stark, 2010).

This is also true in the context of science. Studies have shown considerable gender differences in the visibility of scientists (so-called ‘Gender Visibility Gap’, Klammer and Wegrzyn, 2023). These differences exist at two levels of visibility: within science, for example, in publications and citations (e.g. Chatterjee and Werner, 2021; Huang et al., 2020; Van den Besselaar and Sandström, 2017), and in science communication (e.g. Eizmendi-Iraola and Peña-Fernández, 2023; Fletcher et al., 2021; Prommer and Stüwe, 2020), that is, the transfer of research results to society.

This study examines the visibility and representation of female scientific experts compared to their male counterparts on the second level (in science communication), namely in German news media coverage of eight science-related risk issues (the regulation of glyphosate, the dangers of nitrogen oxides and dioxin, COVID-19, Ebola, flu pandemics, antimicrobial resistance and the legalization of marijuana). To guide our analysis, we pose the following overarching research question: How visible are female scientific experts in comparison to male experts in German media coverage across diverse science-related issues? While this central question frames our inquiry, a deeper exploration involves examining specific dimensions, such as variations in the quotation of female and male scientific experts and the contextual factors influencing the selection of scientific experts, including their scientific reputation or hierarchical position.

Our approach is distinctive in several ways. First, we answer our research questions from an issue-comparative perspective (rather than through a single case study or an issue-independent expert study). Second, we compare the representation of female and male scientists in media coverage with extra media data^1^ (Rosengren, 1970), namely their representation as so-called contributory experts^2^ (Collins and Evans, 2002) in corresponding scientific research fields (scientists who publish peer-reviewed papers in the relevant research fields) and their hierarchical positions within the scientific system. Third, we take the gender of the reporting journalists into account and test for potential differences in expert selection between male and female journalists. Fourth, we compare the bibliometric profiles of the male and female scientists referenced in media coverage with those of the male and female contributory scientists.

## 2. Theoretical background, state of research and hypotheses

### Gender representations in media reporting

Theoretically, our study connects to the perspective of the so-called equality approach (Lünenborg and Maier, 2013), which generally demands that women and men have equal opportunities and can equally participate in society. Therefore, it focuses on the analysis of the unequal treatment of women in various areas of society, for example by determining the underrepresentation of women in media coverage. Another theoretical linking point is the news bias approach, which examines imbalances in media coverage, assuming that journalists do not merely reflect reality but present it with bias. Influencing factors include the political stance of the medium and journalists’ own opinions, attitudes and beliefs, among others (Eveland and Shah, 2003). While most studies focus on political bias, news bias research also addresses apolitical biases, such as gender bias (e.g. Davis, 1982; Van der Pas, 2022).

Gender diversity in news media coverage has been a research topic for decades (in Germany, since the 1970s; Küchenhoff and Bossmann, 1975). However, gender representation in *science reporting* became a focus much later (e.g. Huber, 2014; Niemi and Pitkänen, 2017; Prommer and Stüwe, 2020; Soley, 1994). Overall, research consistently shows that women are less frequently cited as actors or (scientific) experts in media coverage than men – even relative to their representation in science – indicating media bias. This holds true even in highly developed countries with strong gender equality rankings (Matthes et al., 2016). And it applies to media coverage in general (e.g. Huber, 2014; Niemi and Pitkänen, 2017; Soley, 1994; Vogler and Schwaiger, 2023; Zoch and VanSlyke Turk, 1998) as well as to science reporting in particular (e.g. Eizmendi-Iraola and Peña-Fernández, 2023; Fletcher et al., 2021; Ioannidis et al., 2021; Joubert et al., 2022; Kitzinger et al., 2008; Prommer et al., 2019; Prommer and Stüwe, 2020). Over time, the imbalance of gender representation has only moderately improved in favour of women (e.g. GMMP, 2020; Huber, 2014). These findings have prompted criticism of journalism (keyword ‘media sexism’), which allegedly disadvantages women in terms of their public visibility (Haraldsson and Wängnerud, 2019; Prommer et al., 2019).

However, the proportion of women in media coverage varies by topic, with a narrower gender gap in human interest stories compared to sports or political reporting, for example (Desmond and Danilewicz, 2010; GMMP, 2020; Prommer and Stüwe, 2020; Schwaiger et al., 2021). For science reporting in particular, recent analyses indicate that women account for approximately 30%–35% of actors in COVID-19 coverage and around 30% in science and health news overall (Berggren, 2020; Fletcher et al., 2021; GMMP, 2020). Therefore, we hypothesize:

*H1.* News media coverage of science-related issues is dominated by male scientific actors.

To demonstrate that the proportion of female actors in news media is biased compared to reality and that women are underrepresented, most studies compare their media presence with real-world data, often using official statistics. For example, studies by Prommer and Stüwe (2020), Kitzinger et al. (2008) and Fletcher et al. (2021) compare the proportion of female actors represented in the media with statistical data on the actual share of women in the respective research fields or among practicing physicians or STEM professionals. They find that these reference values generally exceed – sometimes more, sometimes less clearly – the proportion of female scientists represented in media coverage. Theoretically, this gender visibility gap can be explained either by a demand-side explanation – female scientific experts are approached less frequently by journalists – or by a supply-side explanation, suggesting that when they are approached, female experts are less willing to accept or respond to media requests (Sievertsen and Smith, 2025). We thus hypothesize that female scientists not only appear less frequently than their male counterparts in science-related news coverage, but are also underrepresented relative to their actual presence in the respective research fields:

*H2.* Female scientific actors are underrepresented in news media coverage of science-related issues compared to their representation as contributory experts in the related research areas.

### Gender and scientific reputation as ‘expert factors’

Gender is just one characteristic by which scientific actors selected as experts for media coverage can be differentiated. Several studies analyse or describe typical characteristics that influence the journalistic selection of scientific experts (e.g. Goodell, 1977; Joubert et al., 2023; Olesk, 2021). Nölleke (2013), drawing on news value theory (Galtung and Ruge, 1965), distinguishes various ‘expert factors’ that determine the value of an expert for journalists. These factors consist of (more or less) objectively determinable factors specific to the person (e.g. age, gender or scientific reputation), media-specific factors (e.g. previous media experience and visibility or attractive appearance), content factors (e.g. communication skills or strength of opinion) and interactional factors (e.g. availability or reliability). Within the scope of our quantitative content analysis, we can only measure those factors that can be extracted from the article text (such as gender) or publication databases (such as scientific reputation). We consider scientific reputation^3^ to be a particularly relevant factor to include in our analysis, as from the perspective of (normative) theories of the public sphere,^4^ a disconnect between scientific reputation and media visibility poses significant concerns, particularly when the scientific knowledge shaping media coverage predominantly originates from individuals lacking recognized scientific reputation in the field.

Recent studies on the relationship between scientific reputation and expert selection in German news coverage suggest a positive correlation between scientists’ publication records and their likelihood of being selected by journalists, particularly in specialized science departments (Lehmkuhl and Leidecker-Sandmann, 2019; Leidecker-Sandmann et al., 2022). However, earlier research (e.g. Goodell, 1977; Shepherd, 1981) and a recent study by Ioannidis et al. (2021), which apply different methods and criteria,^5^ do not confirm this link.

Thus, the findings remain inconsistent, and it is unclear whether the scientific reputation of public experts differs by gender. It is conceivable that female scientists must achieve a comparatively higher level of scientific reputation to be considered for journalistic selection. This assumption aligns with several studies (e.g. Fletcher et al., 2021; GMMP, 2020; Ioannidis et al., 2021; Prommer et al., 2019; Prommer and Stüwe, 2020), which suggest that female scientists often face higher barriers to media visibility, requiring exceptional scientific reputation to overcome structural and perceptual biases. We formulate the following open research questions:

*RQ1a.* Do scientific actors visible in media coverage have a relatively higher scientific reputation compared to their non-visible scientific colleagues?
*RQ1b.* Is this difference more pronounced for female or male visible scientific experts?

### Influence of the journalists’ gender

In addition, there are studies that consider the potential influence of the journalists’ gender on the selection of male or female experts for media coverage. For example, Zoch and VanSlyke Turk (1998), Huber (2014) and GMMP (2020) show that female journalists are significantly more likely than male journalists to reference female experts or sources.

One theoretical approach that helps explain this phenomenon is the homophily principle, which suggests that similarity breeds connection. It describes the tendency of individuals to surround themselves with others who share certain characteristics, such as gender, age, or social norms (McPherson et al., 2001). As a result, male journalists may unconsciously select male experts, either because they identify more strongly with them or because these experts are more accessible through personal networks or because of shared professional circles.

Another relevant framework is the role congruity theory (Eagly and Karau, 2002), which posits that social norms and expectations shape individuals’ behaviour based on gender. Since attributes such as competence, authority and rationality are often associated with men, while women are more commonly linked to traits like empathy and emotionality (Eagly and Karau, 2002), female scientists may be perceived as less prototypical or credible representatives of expert roles. This perceptual incongruity may result in a preference for male scientists in media coverage and contribute to the underrepresentation of female scientists in journalistic selection processes, even when their qualifications are equivalent to those of their male peers. Therefore, we assume:

*H3.* The gap between references to female and male scientific actors in media coverage of science-related risks will be smaller in articles authored by women than in articles authored by men.

### Differences in the content of statements

Finally, we examine the content of statements from female versus male scientists. Some studies suggest that female scientific experts tend to express themselves more cautiously and strive to appear as neutral and objective as possible in public discourse, and are less likely to make action-related (political) recommendations (e.g. Niemi and Pitkänen, 2017; Shine, 2022). A possible theoretical explanation for this phenomenon is again role congruity theory. According to this framework, women are often expected to exhibit traits such as restraint and cooperation, whereas men are associated with authority, assertiveness and decisiveness (Eagly and Karau, 2002). These gendered expectations may influence how women communicate in public settings, prompting them to adopt a more cautious and neutral tone to avoid challenging traditional femininity stereotypes. Therefore, we hypothesize:

*H4.* Female scientists referenced in media coverage less often communicate (political) recommendations within their quoted statements than male scientists.

## 3. Data and method

### Issue selection

To test our hypotheses and answer our research questions, we present a secondary analysis of the results of several standardized quantitative content analyses that have been carried out over the past 10 years using the same coding scheme^6^ as part of the ‘Science in Public Monitoring Project’. In this project, we examine the representation of different groups of social actors in journalistically mediated public discourses on an ongoing basis. So far, we have conducted content analyses of eight science-related risk issues: COVID-19, antimicrobial resistance (AMR), Ebola, flu pandemics, regulation of glyphosate, dioxin and nitrogen oxides (NOx) as well as the debate on the legalization of marijuana. These issues were selected purposefully. Our aim was to select various science-related issues that have attracted focused media interest at least once in Germany in the last 30 years. All phenomena also have in common that they (1) pose health risks, (2) revolve around social and legal decisions and (3) are the subject of scientific research and scientific knowledge is required to explain, understand and assess these risks.

The results of our content analyses allowed us to categorize these issues based on the prominence of scientific experts in media coverage. The debates on AMR, Ebola and two potential pandemic flu outbreaks in Germany – swine flu (2009) and bird flu (2005/2006) – are characterized by a strong presence of scientific experts, whereas political actors were less prominent in the media coverage. In all three cases, scientific experts account for more than 40% of all references in the analysed media articles, leading us to classify them as *science centred* debates.

In contrast, four other debates – on glyphosate regulation, dioxin, nitrogen oxides and marijuana legalization – place much less emphasis on scientific expertise. These issues are dominated by political actors and revolve around concrete policy decisions in which scientific input serves primarily as a supporting element. References to scientific experts account for less than 20% of all references. Accordingly, we call this issue group *policy centred* debates.

The media debate on COVID-19, our eighth issue, stands out as exceptional in many respects (e.g. the volume of coverage) and should therefore be considered separately from both issue groups. Based on the criterion used here, this debate was not dominated by scientific experts in purely quantitative terms. Instead, representatives of the political executive and representatives of particular interests played a prominent role in COVID-19 media coverage (Leidecker-Sandmann et al., 2022). However, scientific expertise had a significant qualitative impact, serving as a key reference point for justifying political decisions, most notably lockdown measures. This underscores the crucial role of scientific experts in shaping policy, even when their presence in the overall media discourse was less prominent. Combined with the sheer volume of media reporting, this created the general impression that scientific experts were more visible in the COVID-19 pandemic than ever before.

### Media title selection

We analysed the coverage of the eight issues mentioned above in four German media titles: the national daily newspapers *Die Welt* and *Süddeutsche Zeitung*, the weekly news magazine *Der Spiegel* and the news agency *dpa*. Our analysis focuses on these national quality news media because the *Süddeutsche Zeitung* (left-liberal), *Die Welt* (conservative) and *Der Spiegel* (left-liberal) are influential opinion-shaping outlets which rank among the largest and most influential in Germany. They are widely read by political and business leaders as well as journalists and are therefore considered ‘leading media’. The *dpa* is Germany’s largest news agency and serves as a key source and pacesetter for news, particularly within the regional press. We collected corpora of thematically relevant articles via keyword searches (see Supplemental Materials) in the databases *wiso presse* (*Die Welt* and *Der Spiegel*), *SZ LibraryNet* (*Süddeutsche Zeitung*) and *dpa-news.de* (*dpa*).

### Data basis content analysis (visible scientists)

Our detailed analysis is based on a total of 4860 articles that appeared between 1995 and 2020, drawn from stratified random samples (exact descriptions of the sampling procedure are provided in the Supplemental Materials). Only a specific section of the total data material (of which Table 1 provides an overview) is relevant for this study, namely references to individual scientific experts. In the 4860 articles, we identified approximately 13,000 references to different actors, of which almost 8800 are attributable to individual actors, with the rest attributed to organizations such as the WHO and Greenpeace. A reference is defined as a passage in which one or more statements of an actor are quoted (directly or indirectly). Of these 8800 references, approximately 1800 relate to individual scientific experts (20%), and 1500 relate to experts from the life sciences. They come from the 1124/904 different scientific experts at the centre of this study.

**Table 1.** Data basis per issue.

	Articles	References (all)	References to individual scientific experts	Actors (individual scientists)	References to individual life scientists	Actors (individual life scientists)	Investigation period
**Science centred debates**							
AMR	152	358	126	92	116	84	1 January 1997–31 December 2013
Pandemic flus	175	534	166	107	161	102	1 January 1995–31 December 2015
Ebola	330	991	171	100	164	96	1 January 1995–31 December 2015
**Policy centred debates**							
Marijuana	234	693	64	40	51	31	1 January 2012–31 May 2019
Glyphosate	298	609	71	42	65	36	1 January 2015—31 December 2019
NOx	489	1183	166	94	122	68	1 January 2011–30 June 2019
Dioxin	997	2319	229	153	188	124	1 January 1999–31 December 2019
**COVID-19 debate**							
COVID-19	2185	6382	776	496	609	363	1 January 2020–31 December 2020
**All**	**4860**	**13069**	**1769**	**1124**	**1476**	**904**	**1 January 1995–31 December 2020**

‘Articles’ refers to the total number of analysed articles on each issue. ‘References (All)’ is the number of references made to all actors (be it individual, institutional, or generic actors) within these articles. ‘References to individual scientific experts’ is the number of ‘References (All)’ attributable to individual scientific experts. ‘References to individual life scientists’ is the number of references to ‘individual scientific experts’ who can be assigned to the field of life sciences. ‘Actors (individual scientists)’ is the number of individual scientific actors that are referenced in the article (each actor is counted only once, no matter how often they are referenced). ‘Actors individual life scientists’ is the number of individual scientific actors belonging to the research field of life sciences (each actor is counted only once).

### Data basis control sample (contributory experts)

In addition, we have gathered *extra media data* by analysing a random sample of 2400 actively publishing scientific experts in the respective fields of research (300 experts per issue). The prerequisite for inclusion in this sample was the publication of at least one scientific paper as first or last author (in which at least one person with German affiliation^7^ was involved) between 1995 and 2021 in research areas corresponding to the eight analysed issues. More specifically, the control sample was constructed in four steps. First, we compiled a comprehensive list of all publications with a German institutional affiliation in the relevant research areas using the Web of Science database. Second, we extracted the names of all first and last authors from these publications, as they typically represent the most visible contributors to scientific work. Third, we merged multiple spellings or variations of names referring to the same individual (name disambiguation). Finally, we randomly selected 300 scientists per issue area to form a control group for comparison.

The life scientists in the resulting list are what Collins and Evans (2002) call contributory experts and represent our *control sample*.^8^ From this control sample, we derive the proportion of female and male researchers who actively publish in a specific research area. In addition, we determined the scientific reputation of all contributory experts and estimated the share of male and female researchers in scientific leading positions in the respective research fields.

### Levels of analysis

We distinguished two different levels of analysis: the *individual actor level* and the statement or *reference level* in order to study not only the representation of individual experts but also the reference pattern. At the actor level, each person is counted only once, regardless of how often they are referenced. For the reference level, we count each reference, so one person can appear multiple times. The actor level analysis includes bibliometric comparisons with the control sample and therefore had to be restricted to the 904 life scientists. For other scientists such a comparison is not feasible. The reference level analysis is not restricted to life scientists. It includes references to all 1124 visible individual scientific experts.

### Coding and measures

For each actor, we coded the *name, institutional affiliation* and *affiliation to a societal area* (such as political executive, partial interest group, science).^9^ For all scientific actors (including the scientists from the control sample), we additionally coded the *gender* – our central variable – automatically using the platform *Namsor* (https://namsor.app/). In addition to a binary gender classification for a name, *Namsor* returns a certainty for this classification in form of a value between 50% and 100%. Based on the results of a manual validation, we are confident that the classification is very accurate when the certainty is above 90%.^10^ All names for which the certainty was below this threshold or for which only initials were available were coded manually. Here, the procedure was as follows: first, an attempt was made to make a classification based on the name and personal knowledge of whether a name is more commonly used for male or female persons. If coders were unsure, they searched for the scientist’s name using Google and looked at up to three hits to arrive at a classification. The gender of the *authors of the articles* was classified manually using a category with three values (male, female, other/not known;^11^ determination based on the first name).

For the analysis of the references, we distinguish between three *types of statements* (based on the decisionist model of political advice; Weber, 1922), namely substantiations (e.g. ‘we had X nosocomial MRSA infections last year’), risk interpretations (e.g. ‘the situation is worrying/not dramatic’) and action-related political or individual recommendations (e.g. ‘shops should remain closed’, ‘you should wash your hands more often’). We have further differentiated these statement types in terms of their content. We differentiated the action-related statements according to the addressee of the request (politics, civil society or individuals), the substantiations according to how precise they are and the interpretations according to whether they are more alarming or reassuring.

To assess the *scientific reputation* of the visible scientists, we collected the bibliometric profiles of all life science experts who were mentioned by name in the articles or who were part of the control sample. We used the *PubMed Europe* database, which primarily contains biomedical publications. This is why experts from other fields have not been analysed. Publication data was collected with the help of a Python script that automatically retrieves the data from the *PubMed Europe* API (Milhahn et al., 2018).^12^ In our analysis, we focus primarily on productivity and impact, operationalized by the total number of publications in Q1-Q3 journals and the h-index.^13^ Q4 journals were excluded because they are unlikely to be relevant for reputation building, at least for German life scientists.

For each issue, we compared the bibliometric indicators of the visible scientists with those of the control sample of 2400 experts. In addition, we used the publication profiles to identify scientists in leading positions. We operationalized leading positions via the order of authorship: scientists who appeared at least three times^14^ as last-named authors were defined as scientists in senior roles as it is common practice, particularly in the hard sciences, that the leader of a research group is credited with last authorship (Müller, 2014).

### Data collection

Data were collected over several years, the first analyses being conducted in 2014 (AMR, Ebola, Influenza) and the last in 2021 (COVID-19), with 13 coders generally coding individual topics. The coders had all participated in several training courses held by the project leader, during which the coding of the categories was taught both theoretically and practically, using the same training material (articles that were not part of the analysis). After each meeting, coders practised coding the categories independently; their results were then discussed and compared together at the next meeting. The project leader also participated in the trial coding in order to check the coding not only for consistency but also for validity. When the reliability in these trial codings reached a satisfactory level, a sample of about 20 articles was used for the final reliability test in which the project leader participated as the master coder against whom all coding was tested. These articles were selected randomly and are part of the analysis corpus. In this test, all coders reached satisfactory to very good results for the variables at the actor level (Krippendorff’s alpha: .73–1).^15^

## 4. Results

### Representation of female life scientists in news media coverage

#### Basic case number analysis

**Figure 1.**
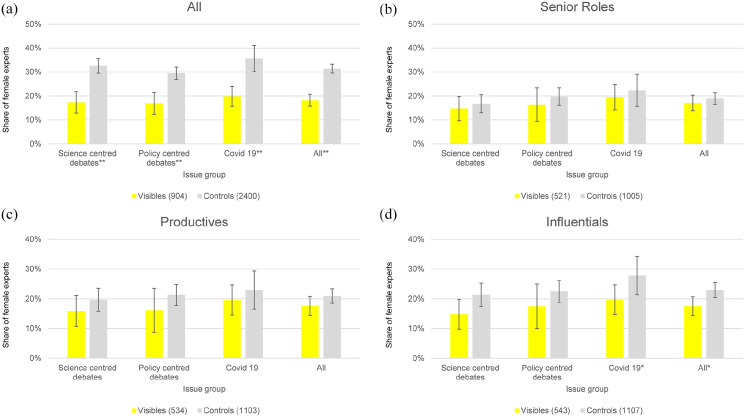
Female shares of visible experts compared with female shares of a random sample of contributing experts by issue group. Panel a shows the comparison for all experts, panel b only includes experts in senior roles and panels c and d are limited to the most productive respectively most influential experts from each group. Error bars show (95% CI)confidence intervals, asterisks indicate significance levels (*p < .05; **p < .001) in a χ2 test 95% .

Simple frequency counts at the actor level show that news media coverage of science-related issues is dominated by male scientific experts (82% vs 18% female scientific experts), confirming H1 (χ^2^(1) = 364.47; *p* *<* .001). However, whether female scientists are actually *underrepresented* in media reporting compared to their representation in relevant research areas (H2) can only be determined by comparing these proportions with those in our control sample of contributory scientists in the respective fields of research. This comparison shows that female life science experts are indeed significantly underrepresented in public media debates compared to their share of actively publishing scientists in the respective fields (18% vs 31%; χ^2^(1) *=* 55.7; *p* *<* .001). This underrepresentation applies to all eight individual issues (Figure 1a).

#### Analysis accounting for control variables

While this simple comparison confirms our second hypothesis, we would like to test the robustness of this finding through further, more differentiated analyses, including control variables based on the characteristics of the scientific experts. For these differentiated analyses, we harmonized our two groups of scientific experts (visible scientists and control sample) with regard to three characteristics: (1) we only compared those who work in *senior roles* (Figure 1b). In addition, we analysed the (2) *most productive* (Figure 1c) and the (3) *most influential* scientists separately (Figure 1d). These groups include those who have published more than half of all others (most productive) or whose papers have been cited more frequently than those of half of all others (most influential).

The difference in the proportion of female experts between the visible scientists and the control sample is then significantly reduced to two to five percentage points. However, although women in senior roles are only slightly underrepresented (Figure 1b) (χ^2^(1) *=* .85; *p* *=* .36), the difference is systematic across all three issue groups. The difference is somewhat greater across all issues when comparing only the most productive (Figure 1c) (χ^2^(1) *=* 2.5, *p* *=* .11) and the most influential scientists (Figure 1d) (χ^2^(1) *=* 6.5, *p* *=* .01). It follows that the large difference in the proportion of women in media coverage is primarily due to the group of experts who do not hold senior positions, have published comparatively few papers and have a comparatively low impact.

### Reputation of female and male life scientists in news media coverage

#### Issue-based reputation analysis

In the next step, we want to analyse the relationship between reputation and public visibility to answer our research questions (RQ1a and RQ1b). For this task, we compare the visible female scientists with the control group of female scientists as well as the visible male scientists with the control group of male scientists according to their productivity (number of publications in Q1-Q3 journals) and impact (h-index).

**Figure 2.**
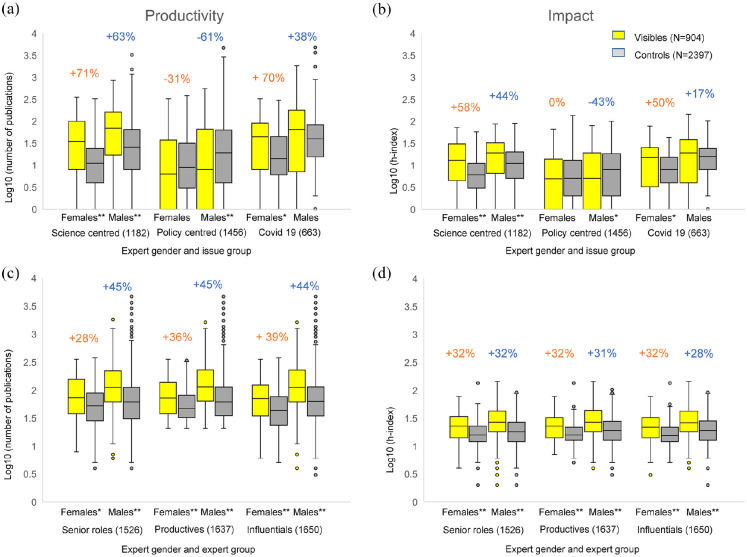
Boxplots of productivity and impact of female and male experts by issue and expert group. Panels a and b compare the productivity (number of publications) respectively impact (h-index) of all visible experts and members of the control group for each issue group. Panels c and d combine data from all issue groups and compare specific subgroups of the visible experts and control group. N﻿umbers above the box plots indicate the reputation gap by gender in percent. Y-axes are logarithmic, asterisks indicate significance levels (*p < .05; **p < .001) in a Mann–Whitney *U* test.

We conduct these comparisons on a group-by-group basis, organized according to issue (Figure 2a and b). This comparison of the issue groups shows that visible scientists of both genders have a significantly higher reputation (productivity as well as impact) than the corresponding control group in the science-centred debates as well as in the COVID-19 debate (Mann–Whitney *U* test *p* *<* .001^16^). Thus, in terms of productivity and impact, visible scientists have a significantly higher reputation in only four of the eight issues.^17^

#### Analysis accounting for control variables

In a second step, we make the same comparisons again, but focus on scientists in senior roles, the most productive and the most influential scientific experts (Figure 2c and d). Our analyses show that across all issues, the visible scientists of both genders in these subgroups (those with senior roles, the most productive and the most influential) have a significantly higher reputation than the control group (Mann–Whitney *U* test *p* *<* .001).

#### Reputation gaps between visible experts and control groups

For a differentiated analysis, we determined the ‘reputation gap’ (Figure 2a to d) between the visible experts and the respective control groups for each gender. For the calculation of the reputation gap, we determined the medians of the number of publications and h-indices as a basis and set them in relation to each other. The basis for the comparison is always the control group. If the group of visible experts has a median of 10 publications and the control group a median of 15, the gap between the two groups is −33%. These differences provide information on whether the reputation gap between visible scientists and the control group is greater (or smaller) for women than for men. Based on this comparison, we can therefore estimate whether visible women have a higher or lower reputation gap compared to visible men, with the respective control group serving as a reference.

Such a comparison is preferable to a direct comparison between male and female scientists because this would require taking into account various control variables that were not recorded in this study. As shown in Figure 2, women have lower scores than men in every category.^18^

The particularly robust comparison of the three subgroups of the most productive, the most influential and the scientists in senior roles indicates that visible female experts differ slightly from men in terms of productivity at best (Figure 2c and d). However, we also find differences in the three issue groups with regard to this characteristic. In the science-centred debates, the gap in productivity and impact between visibles and controls is approximately the same for both women and men (Figure 2a and b). But we find a difference in the expected direction in the policy-centred debates and in the COVID-19 debate.

### Reference patterns

So far, we have looked for differences at the level of the individual actors referenced by journalists. Now we aim to investigate whether journalists reference individual experts differently based on their gender. In addition, we seek to examine whether the gender of journalists influences the referencing of female experts. Accordingly, in the next step of the analysis, we change the unit of analysis (from actors to references). Unlike in the step before, we are no longer restricted to life scientists only and include all references to scientific experts in the analysis. This increases the number of visible experts from 904 to 1124 (Table 1).

#### Basic mean value analysis

**Figure 3.**
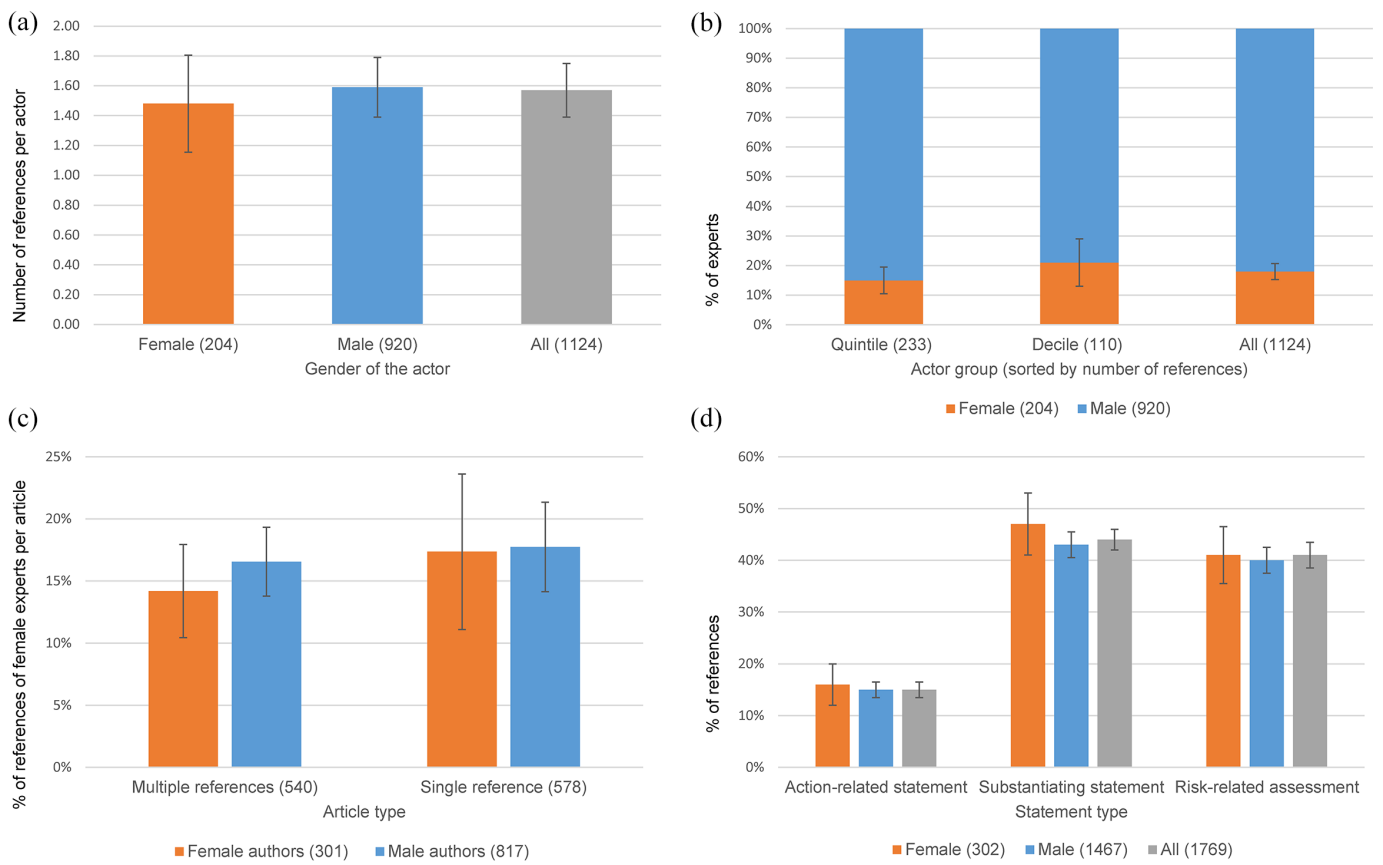
Comparison of referencing patterns of female and male experts. Panel a shows the average number of references for female and male experts, panel b presents shares of female and male actors among the 20 respectively 10% most visible experts. Panel c compares the shares of female experts in articles with only one or multiple references and panel d shows the frequency of different statement types depending on the gender of the actor. Error bars indicate 95% confidence intervals. Note: for color figure please see online version.

In order to uncover potential differences, it is first relevant whether there is a difference between the female share at the actor level (18%; *N* = 1124) and the female share at the reference level (*N* = 1769), which is 17%.^19^ Thus, female experts are slightly less frequently cited than male experts. On average, each female expert is cited 1.5 times; male experts are cited slightly more, namely 1.6 times (*F*(1,1070) *=* .30; *p* *=* .58) (Figure 3a). The gender of the selecting journalist only has a small influence on this characteristic; male authors cite female experts almost as often as female authors (1.4 times vs 1.7 times on average; *F*(1,202) *=* .96; *p* *=* .33).

#### Analysis accounting for citation frequency

However, mean value comparisons are not sufficient in themselves to describe the referencing practice in journalism adequately because the references are quite heavily skewed to the right. The vast majority of experts in our sample were only quoted once, only around 20% were quoted somewhat more frequently, and some were quoted very frequently during the COVID-19 debate. In order to draw a differentiated picture, we have grouped the referenced scientists according to whether they are quoted more frequently than 80% of all other experts (the upper quintile). In addition, we identified the most frequently referenced experts who were cited more frequently than 90% of all other experts (the upper decile). We find that female scientists are slightly underrepresented in the top quintile (15%), but slightly overrepresented in the top decile (21%) (Figure 3b).

#### Analysis by journalist gender

The comparison of whether female journalistic authors differ from male authors in their referencing patterns does not yield a significant result for this attribute either. Female authors cite the upper quintile of female experts 4.7 times, male authors 3.5 times (*F*(1,32) = 1.0; *p* *=* .32).

To sum up, we cannot find any reliable evidence that individual female scientists are on average referenced less often than men. The comparison between female and male journalists does not yield a significant result either. Therefore, we have to reject H3.

#### Analysis by number of expert references per article

Finally, we examined whether the number of expert references in an article affects the referencing pattern, but found no notable differences (Figure 3c). Female expert voices are similarly represented in both longer background articles with multiple expert references (18%) and articles citing only one expert (16%) (*F*(1,1259) = .43; *p* *=* .52), which corresponds to the overall female share of references (17%). In addition, no significant differences were observed based on the author’s gender (χ^2^(1) = 1.47, *p* *=* .23). Female scientists accounted for 18% of all references in articles written by male authors and 15% in articles written by female authors.

### Content of statements

Any gender-related differences in what the experts say have not yet been taken into account. For the quantitative structural analysis of the content of expert references, we distinguish between substantiations, risk interpretations and action-related political or individual recommendations (see ‘Coding and measures’ and Supplemental Materials). As Figure 3d^20^ shows, the structure of the statements with which the experts appear in public does not differ between the genders. Both groups of experts are reluctant to make action-related statements (in total: 15%; by female scientists: 16%; by male scientists: 15%); the majority of statements are substantiations (in total: 44%; by female scientists: 47%; by male scientists: 43%) or risk-related interpretations (in total: 41%; by female scientists: 41%; by male scientists: 40%). We have further categorized the action-related statements based on the target audience of the request (politics, civil society or individuals), classified the substantiations by their level of specificity, and evaluated the interpretations to determine whether they tended to be more alarming or reassuring. This detailed analysis also revealed no evidence that the statements of the female experts differed from those of the male experts (refuting H4). Accordingly, the way scientific experts are quoted publicly does not seem to be affected by their gender.

## 5. Summary and discussion

### Female scientific experts are underrepresented, but context matters

Our analysis reveals that female scientific experts are less visible compared to their male counterparts in German media coverage (18%; H1). While this figure aligns with the proportions reported by Huber (2014) and Soley (1994), it is significantly lower than the 30%–35% found in Berggren (2020), Fletcher et al. (2021) and GMMP (2020). This discrepancy may stem from the fact that these studies consider women among all actors in science and health coverage (GMMP, 2020) or as general experts in COVID-19 coverage (Berggren, 2020; Fletcher et al., 2021), rather than specifically focusing on scientific experts as we did. From an equality perspective, it is evident that achieving a balanced 50/50 representation in both the media and the scientific sphere remains a distant goal.

Regarding the question of whether female scientific experts are also *underrepresented* – indicating a media bias – when assessed against normative reference values other than a strict 50/50 representation, our comparisons with the control sample of contributory experts suggest that the more differentiated the level of comparison, the smaller the differences become, although they do not disappear. Even after controlling for hierarchical position and reputation, the proportion of visible female life science experts remains systematically below research community benchmarks, pointing to imbalances in media coverage. However, these differences are generally small and within the range of random statistical fluctuations. While our findings confirm H2 by demonstrating the underrepresentation of female experts in the public sphere, they also temper the severity of the issue, challenging the notion of pervasive ‘media sexism’.

### Journalism reproduces structural inequalities

Our examination of possible discrimination against female scientific experts through journalistic selection routines, which could be explained by the homophily principle, yielded largely negative results. Female experts are not quoted significantly less often by male journalists than by female journalists (refuting H3); the focus on individual, particularly visible experts is hardly less pronounced for women than for men; and the content with which they are quoted does not differ from that of men (H4), contradicting our expectations derived from role congruity theory, which posits that gendered social norms lead women to communicate more cautiously and in less assertive ways in order to align with stereotypical expectations of femininity (Eagly and Karau, 2002). Female experts also do not need to be relatively more productive or relatively more influential compared to the relevant scientific community than men in order to be referenced by the German quality press. However, in policy-centred debates and in COVID-19 coverage, the gap in productivity and impact between visible scientists and the control sample is more pronounced for women than for men. This may indicate that different selection dynamics may take effect for women than for men depending on the issue.

Our analysis of referencing patterns in the German quality press finds no clear evidence of discriminatory practices in journalism, challenging studies suggesting media sexism or gender bias (e.g. Haraldsson and Wängnerud, 2019). Instead, our findings indicate that hierarchical position, productivity and research impact matter more than gender. Since fewer women hold high-ranking academic positions, publish less frequently and receive lower impact, they are cited less often in science reporting (Huber, 2014). In short, journalism largely reflects structural inequalities in academia. On the downside, we find no evidence that journalistic sourcing practices significantly mitigate structural gender inequality. (Mass) Media do not only depict realities of society – they also (help to) construct them (Tuchman, 1978). The overrepresentation of male scientific experts and the relative underrepresentation of female scientists in press coverage both mirror and reinforce existing biases, influencing perceptions of credibility and authority in science. A more balanced representation could challenge these patterns, foster a more inclusive understanding of expertise and potentially boost women’s confidence in entering male-dominated research fields. For instance, a cross-country analysis shows that greater female representation is positively linked to women’s political interest and perception of politics’ importance (Bühlmann and Schädel, 2012).

### Significance of the visibility of female experts

Our findings contribute to the literature in at least four ways. First, by going beyond simple frequency counts and integrating bibliometric and contextual data, our study shows that expert visibility in the media cannot be fully explained by individual-level academic credentials alone. Second, our issue-comparative approach reveals that selection dynamics are context-dependent: in policy-centred debates, female experts appear to face higher barriers to visibility even when their reputational credentials match those of male colleagues. Third, the lack of meaningful gender differences in expert statements and citation frequency challenges assumptions about communicative or stylistic gender gaps, emphasizing the relevance of structural constraints. Fourth, our findings provide little indication of active media sexism – that is, of journalism playing a direct role in producing or amplifying gender-based inequalities.

By recognizing media as an active force in shaping public understanding, this study highlights the need to examine how gendered expert visibility may influence perceptions of science and gender equity. Addressing these patterns can promote a more inclusive public discourse, which is essential for informed decision-making and societal progress. Our findings underscore the need to critically reflect on the systemic filters through which expertise is made visible. Journalism plays a crucial role in shaping public perceptions of who counts as a credible scientific voice. A more diverse composition of visible scientific experts – without compromising on credibility – could contribute to more balanced and inclusive representations of scientific perspectives.

## 6. Limitations and directions for further studies

Several limitations of our study should be noted. For instance, based on our analysis, we can only make descriptive statements for the visibility of female scientific experts within a small part of the German print media coverage, so our findings cannot simply be transferred to other media titles, genres, or countries, which should be addressed in future studies. Further, our sample of visible experts is not a homogeneous group of researchers. The proportion of functionaries in science-related (non-)governmental organizations, such as heads of department, senior physicians and heads of local health authorities, is comparatively high (especially in the group of visible experts with no senior position, few publications and low impact, namely around two thirds). Thus, our sample consists of scientists who work at universities or other research institutions as well as many managers in governmental and non-governmental organizations. Their public spokesperson role is thus not always primarily legitimized by their scientific expertise, but by their professional role in a non-scientific context. An example is the *Robert Koch Institute*, which is a federal government agency attached to the *Ministry of Health* that also has research tasks, but essentially fulfils specialized administrative and supervisory tasks. Because of the high proportion of functionaries in the group of those visible experts with comparatively few publications, a comparison with the control group of contributory experts appears to be at least distorted.

Furthermore, in the context of this study, we cannot answer which factors are relevant for the (non-)selection of female scientists. However, we assume that the lack of evidence for systematic journalistic discrimination against female scientists points to the somewhat stricter self-selection of female experts, as suggested by some studies (e.g. Howell and Singer, 2017; Shine, 2022; Sievertsen and Smith, 2025). For example, a survey of expert economists by Sievertsen and Smith (2025: 454) shows clear evidence of a ‘gender gap in willingness to give an opinion’, noting that ‘male panel members are, on average, 11 percentage points (around 20%) more likely than female panel members to give a substantive opinion on a range of policy topics’. These findings suggest that visibility gaps are not solely the result of media gatekeeping but also reflect deeper social dynamics of self-selection, which could be one possible explanation for the differences documented in our study and an exciting starting point for further studies.

Of course, there are other factors besides gender according to which journalists select experts for media coverage, such as availability and reliability, media experience, or attractive appearance (e.g. Nölleke, 2013). Hence, it is not one criterion alone that makes an actor newsworthy, but rather a bundle of factors (Peters, 2014) which we were unable to take into account in the context of the present analysis. Future studies could examine the decision-making processes within newsrooms, focusing on how editors and journalists select and prioritize scientific experts for coverage. This could provide insights into potential structural biases within the media industry.

## Supplemental Material

sj-docx-1-pus-10.1177_09636625251363937 – Supplemental material for Female expertise in public discourses: Visibility of female compared to male scientific experts in German media coverage of eight science-related issues Supplemental material, sj-docx-1-pus-10.1177_09636625251363937 for Female expertise in public discourses: Visibility of female compared to male scientific experts in German media coverage of eight science-related issues by Melanie Leidecker-Sandmann, Nikolai Promies and Markus Lehmkuhl in Public Understanding of Science

## Notes

1. The term ‘extra media data’ refers to a methodological approach for assessing media content quality by comparing media coverage to external, often statistical indicators, as proposed by Swedish scholar Karl Erik Rosengren (Donsbach, 2008).

2. According to the definition of expertise by sociologists Collins and Evans (2002), an important form of scientific expertise is the ability of scientists ‘to contribute to the science of the field being analysed’ (p. 254), for example through publications. These scientists are the so-called ‘contributory experts’ in the field of research.

3. In this article, we use the term ‘scientific reputation’ exclusively in the sense of scientific productivity and impact, operationalized by the total number of publications in Q1-Q3 journals and the scientist’s h-index. We are aware that other aspects can also contribute to a scientist’s reputation, such as awards and honours (Merton, 1968), public fame or media visibility (Väliverronen, 2021). However, we cannot cover these aspects in our analysis.

4. Theories of the public sphere address the ways in which communication and discourse shape societal dynamics and democratic processes. Rooted in Habermas’ (1992) seminal work, they explore how public discussions – whether through media, institutions or informal networks – mediate between private individuals and broader societal structures. In this context, these theories highlight the importance of ensuring that the information shaping public opinion comes from credible and reputable sources, such as scientists with recognized expertise.

5. Goodell (1977) conducted an in-depth study of seven scientists who were considered highly prominent during the early 1970s. Shepherd (1981) analysed media coverage on marijuana in US-American print media and identified the most cited science authorities. For these, he checked whether they had published any research on marijuana in scientific journals. Ioannidis et al. (2021) compiled a sample of highly visible scientific experts in the COVID-19 reporting in four countries using ‘pre-existing lists of experts who had prominent visibility in media’ (p. 2). They subsequently verified whether these scientists were included among the ‘top 2000 scientists worldwide’ (p. 3) based on their citation impact related to COVID-19.

6. See Supplemental Materials.

7. This is because German journalists tend to prefer quoting German experts. Empirical analyses show that the majority of experts referenced in news articles (about 70%–90%, depending on the study) are from their own country (e.g. Huber, 2014; Niemi and Pitkänen, 2017; Nölleke, 2013).

8. More details on the construction of the control sample including the search strings are included in the Supplemental Materials.

9. Excerpts from the codebook are provided in the Supplemental Materials.

10. Details on this validation study are provided in the Supplemental Materials.

11. Although we are aware that the use of a simple ‘male-female’ category narrows the facets of gender with their respective sub-dimensions, more differentiated classifications were not possible based on the journalistic content. In cases where a media article was written by several authors of different genders, ‘mixed’ was coded.

12. To validate the results, we compared them with manually collected publication profiles of 229 researchers using *Scopus*. The number of publications correlated with *r* = .72 and the h-index with *r* = .77.

13. The h-index is a key metric for the global perception of a scientist in professional circles. It is based on bibliometric analyses of citations to the scientist’s publications. A high h-index results when a significant number of a scientist’s publications are frequently cited in other publications.

14. We set a minimum threshold of three last authorships to more reliably identify scientists in higher hierarchical positions – the more often they appear as last authors, the greater the likelihood that they are group leaders. Our results remain relatively robust to slight variations in this criterion. However, we cannot guarantee that all last authors of scientific publications actually hold senior positions; rather, our approach provides an approximation of the actual distribution.

15. Detailed results from the reliability test are provided in the Supplemental Materials, Table S1.

16. Distributions of bibliometrics are similar for male and female scientists in shape and spread, this is why we interpret the Mann–Whitney *U* test as a test of median differences.

17. These are the four issues in which scientific expertise is of comparatively high relevance (AMR, Ebola, pandemic flu and COVID-19). Accordingly, we only find a positive correlation between reputation and visibility in debates in which scientific expertise can be attributed a significant role. This applies to both women and men.

18. However, this does not necessarily indicate that they have a lower reputation. Direct reputation comparisons between men and women must at least control for differences in career duration and affiliations, which was not possible in this context (Boekhout et al., 2021; Huang et al., 2020).

19. At the actor level, this value indicates the percentage of visible experts that are female; at the reference level, it indicates the percentage of references to female experts.

20. The bars for each gender do not have to add up to 100% because not every actor has to make a statement and a statement can be assigned to several types.
