# Supplementary material for: Female expertise in public discourses: Visibility of female compared to male scientific experts in German media coverage of eight science-related issues
Source: Public Underst Sci. 2025 Sep 7;35(1):2–23. doi: 10.1177/09636625251363937 (PMC12741163; doi:10.1177/09636625251363937)
Supplement: sj-docx-1-pus-10.1177_09636625251363937 – Supplemental material for Female expertise in public discourses: Visibility of female compared to male scientific experts in German media coverage of eight science-related issues [file sj-docx-1-pus-10.1177_09636625251363937.docx]

**SUPPLEMENTAL MATERIALS for**

**Female expertise in public discourses. Visibility of female compared to male scientific experts in German media coverage of eight science related issues**

**Authors**

Melanie Leidecker-Sandmann*, Karlsruhe Institute of Technology, Department of Science Communication, Adenauerring 12, 76131 Karlsruhe, Germany. Contact: [leidecker-sandmann@kit.edu](mailto:leidecker-sandmann@kit.edu). ORCID: 0000-0001-7203-2448

Nikolai Promies, Karlsruhe Institute of Technology, Department of Science Communication. ORCID: 0000-0002-4804-4155

Markus Lehmkuhl, Karlsruhe Institute of Technology, Department of Science Communication. ORCID: 0000-0001-8295-6548

*Corresponding author

**Table of Contents**

1 Excerpts from the codebook (translation from German) – Identification and
 specification of actors in news media articles 1

2 Coding examples (translation from German) 6

3 Excerpts from the codebook (translation from German) – Types of Statements 6

4 Reliability values 10

5 Sampling procedure 10

5.1 Dioxin 10

5.2 Glyphosate 10

5.3 Nitrogen oxids 10

5.4 Marijuana 10

5.5 Ebola 11

5.6 Flu pandemics 11

5.7 AMR 11

5.8 COVID-19 11

6 Formation of the control sample 12

7 Automated gender coding via Namsor 12

# 1 Excerpts from the codebook (translation from German) – Identification and specification of actors in news media articles

*1.1 Identification of actors*

Actors are individuals or organizations that speak directly or indirectly in an article. The actors and what they say form the unit of analysis in this step. In principle, every actor who is quoted directly or indirectly is coded. If two actors appear in connection with a statement, only the first actor is recorded (e.g. "According to the concurring assessments of the WHO and CDC, the situation is threatening").

Make sure that synonymous terms are used relatively frequently for one and the same source. For example: "According to the White House, the risk of resistance has increased massively over the past ten years. President Obama declared yesterday that this is 'worrying'". Or: "Spain and the EU reassured. Prime Minister Rajoy said that the Spanish healthcare system is one of the best in the world..."

In such cases, no two actors are to be coded, but the person named is always to be considered the source. As a general rule, only one actor per organization may be coded, unless two named representatives of one and the same organization express themselves; only in such cases are two (or more) actors belonging to the same organization to be coded (e.g. in an article, the WHO is mentioned ten times as a source and a named representative of the WHO once. In such a case, only the named actor is coded).

If an actor appears with several statements in one article, the following applies: The message of an actor is to be coded, regardless of how scattered these statements appear in an article. In principle, the statement that appears first in an article is coded, with the exception of substantiations; the substantiation with the highest level of precision is coded; if several substantiations of the same level are cited, the first one is to be coded.

Actors who are not quoted directly or indirectly are not coded either. For example, if it is said that Obama has sent aid troops or Anan has appointed someone, the actors are not coded. Actors are only coded if they are mentioned in connection with a statement made by these actors.

- 1. *Specification of the actor*

This variable describes how specifically the actor is described. An actor is any named person, group of people, organization/institution or generally defined collective speaker who is mentioned in an article. We distinguish between three categories:

*1 Individual*

The actor is coded as individual if a specific person is named. In this case, the following information must also be coded: Name, professional position (e.g. hygienist), academic title (e.g. professor), professional affiliation (e.g. Mainz University Hospital, RKI).

Example: "The President of the German Society for General Practice and Family Medicine, Michael Kochen, recommends that German GPs do not vaccinate. "The risk of harm outweighs the benefit," says the Göttingen professor." (Der Spiegel, 19.10.09)

*2 Institutional*

Institutional is coded when only one organization or institution is cited as a source (e.g. WHO, Charité, University Hospital, Chamber of Commerce, The Greens, etc.), but it remains unclear who is actually speaking or making a statement. The name of the organization must be entered as an alphanumeric string.

Example: "One example of such germs is Klebsiella pneumoniae: According to the ECDC, the proportion of its variants that are resistant to carbapenem-type antibiotics among all germs in this group increased in four EU countries from 2009 to 2010" (taz, 18.11.11)

*3 Generic*

The generic category refers to actors that are represented as comprehensively defined generalities. If collective terms such as "experts", "scientists", "biologists", "politicians" etc. are mentioned unspecified as sources of statements, generic coding should be used.

Example: "The greatest fear of epidemiologists: swine and bird flu could form an almost invincible alliance - one brings its high infectiousness to the viral alliance, the other its lethality." (Der Spiegel, 04.05.09)

Note: The three categories are to be considered hierarchically. I.e., if the same actor is mentioned by name ("Prof. Werner Schmidt") and also described by organization or generically ("scientist at Mainz University Hospital", "biologist"), he is to be coded as an individual actor. In the same way, if an article is about "researcher at institution XY", the actor must be coded as institutional if the institution/organization is identifiable.

Example 1: "At the same time, drug experts warn of possible side effects of the serum. According to Drug Telegram editor Wolfgang Becker-Brüser, what is currently underway is a large-scale trial on the German population. New manufacturing processes and new additives that enhance the active ingredient are being used for the drug." (03.08.2009)

In this example, it is a substantiating and risk-related statement that refers to the dangers of the vaccine. In this statement, drug experts are first named and then a specific person is cited as one of these drug experts. Within a statement (determined by actor and topic), the actor must be coded with the highest possible specification category. In this example, Arznei-Telegramm publisher Wolfgang Becker-Brüser is one of the drug experts and must be coded as an individual actor.

Example 2: "After extensive investigations, the experts now assume that the H1N1 virus has also 'prepared the ground' for infection with these germs: 'It has opened the door, so to speak, for the bacteria,' said a spokeswoman for Essen University Hospital." (taz, 09.10.09)

In this example, three actors are recognizable: the experts, a spokesperson and Essen University Hospital. According to the logic of the specification hierarchy, Uniklinik Essen is the most highly specified actor and must be coded as the source of the statement.

- 1. *Name of individual actor*

If an individual actor was coded, the first and last name of the actor must be entered.

- 1. *Institutional affiliation of the actor*

Enter the name of the institution or organization to which the actor belongs (record in full text). For example, this could be the name of a university at which a scientist conducts research or the name of a company. The institution is only coded if it is explicitly mentioned in the article or if it is clear from the actor's (official) title. For example, if the Federal Chancellor is mentioned, you can code "Federal Chancellery" as the institution. If the Minister of Health is mentioned, you can code "Ministry of Health". The institution is coded as precisely as possible (with location). Otherwise (if the institution is not recognizable), this field is left blank (for example, if only "researcher" or "artist" is mentioned).

ATTENTION: When coding the institution/organization, you can be a little more "generous" in that you can enter the institutional affiliation - which should be generally known - for well-known personalities such as the Federal Chancellor, Federal Ministers, EU Commissioners, even if it was not explicitly mentioned, for example: Federal Chancellery, Ministry of Health, European Commission. This information is not always explicitly mentioned in the article, but the average reader - as we like to say and whose impression we are concerned with - knows that Angela Merkel is/was German Chancellor.

- 1. *Affiliation of the actor to a social sphere*

This variable describes a social area to which the actor (speaker of the statement) can be assigned.

*100 Science*

The scientific sphere can be assigned to individual, institutional and generic sources. The category of science encompasses researchers without political, administrative or social functions.

"Scientist", "researcher" or "biologist" are clearly scientific actors. Actors of the DFG are also clearly scientific actors.

Members of the IPCC also count as scientists.

One definition criterion for scientific actors is that they wor independently/ objectively, i.e. not guided by interests in the narrow sense.

ATTENTION I: If it is recognizable that a medical doctor is speaking in his role as a scientist (i.e. researching and not practicing), "100 = science" should be coded. If it is not clearly recognizable, then "400 = medicine" is coded.

ATTENTION II: Employees of university hospitals (except nursing staff, administrative staff), such as chief physicians or senior physicians, are coded as scientific actors with the corresponding scientific discipline "medicine".

ATTENTION III: Employees of private research institutes are also counted as scientific actors - but NOT employees of commercially oriented companies that also conduct research (these are to be coded as partial interest representatives).

*200 Politics*

The political sphere explicitly includes political actors such as government institutions, political administration (e.g. ministries) and political parties. We distinguish between

*210 Political executive*

Includes members of government (EU Commission, federal, state or local). If an actor has several roles (Gröhe was both health minister and party politician (CDU)), the role in which he speaks in the respective context must be coded. If this is unclear, executive actors are always coded as executive. Political executive actors also include the UN.

*220 Political administration (ministries, health authorities)*

Please note: If the actor is a minister of a ministry, they must be coded as a government representative with 210. From the State Secretary level, an actor is coded 220. Actors who belong to the FAO (UN body), UNESCO and the like are also coded as political administrations.

*230 Political legislature*

Members of parliaments at all possible levels, i.e. EU, federal, state, district and local authorities

231 CDU

232 SPD

233 Greens

234 FDP

235 AfD

236 The Left

237 Others

*300 Scientific administration*

Describes the somewhat narrower class of scientific institutions that also perform administrative functions. These include the departmental research institutions already mentioned above, which are subordinate to a federal or state ministry, namely - to name the most important - for example the Robert Koch Institute, the Federal Office for Risk Assessment, the Friedrich Löffler Institute, the Paul Ehrlich Institute or the Federal Office of Consumer Health Protection. These also include international institutions such as the WHO or the ECDC or the American CDC (Centers for Disease Control and Prevention) or the NIH (National Institutes of Health).

*400 Medicine*

Refers to medical professionals, namely doctors, not other hospital staff in general. If a clinic makes a statement, it is coded as medicine.

*500 interest groups*

We distinguish between interest groups (interest group 1) that represent collective goods, such as environmental protection, animal welfare and peace, and interest groups (interest group 2) that represent the interests of specific social groups. Interest groups 1 include Greenpeace, Nabu, WWF and NGOs. No distinction is made here as to whether these are national or international players.

*510 Interest groups1*

These include actors or organizations that represent so-called collective goods interests, such as environmental protection, etc.

*520 interest groups2*

These include trade unions, churches, representatives of commercial enterprises including pharmaceutical companies and the like. Patient organizations are also representatives of partial interests, not collective interests. No distinction is made as to whether these are national or international players.

ATTENTION: Employees of private companies are also classified as partial interest representatives.

*600 Other*

In generic sources, the affiliation of actors must be coded if it is explicitly indicated which field they come from. For example, "scientist", "researcher" or "biologist" are clearly scientific actors. "CDU members" or "EU representatives" are political actors. "WHO experts" is to be coded as scientific administration and "doctors" as medicine. In such cases, if the area of the actor is not recognizable (e.g. if simply "experts" are quoted), these actors are to be coded as other. Even if none of the aforementioned areas appear to apply, but other areas of society that are peripheral in the Habermasian sense are addressed, "600" is coded. Examples include "museums" or "zoos".

# 2 Coding examples (translation from German)

*Example 1:*

“The President of the German Society for General Medicine and Family Medicine, Michael Koch, recommends that German general practitioners not vaccinate. "The risk of damage outweighs the benefit," says the Göttingen professor.” (Der Spiegel, October 19, 2009)

*Coding of example 1:*

Name = Michael Koch

Affiliation = German Society for General Medicine and Family Medicine

Social Sphere = Scientist

*Example 2:*

“The health offices are among the most important control centers when it comes to dealing with the corona pandemic. They record the number of cases, for example, and initiate countermeasures on site. Chancellor Angela Merkel (CDU) now praises the offices for their work. The pandemic is causing tasks there that “result in incredible added value,” she said at the beginning of a virtual conference with representatives of the public health service.” (Der Spiegel, September 8, 2020)

*Coding of example 2:*

Name = Angela Merkel

Affiliation = Federal Chancellery

Social Sphere = Political Executive

# 3 Excerpts from the codebook (translation from German) – Types of Statements

*3.1 Identification of Statement*

The coding unit is the statements within an article.

A statement is any identifiable utterance by actors who have their say in the article

that are semantically linked to one of our science-related risk issues.

Example: "Silke Schwartau: Everything takes time. The study by the environment minister from North Rhine-Westphalia on the use of antibiotics and the revelation by the environmental organization BUND that poultry is infested with resistant germs are not that old. We are also noticing that consumers are becoming more aware of the subject of meat consume." (Die Zeit, 23.02.12)

Example 2: "According to the ECDC authority, in several countries 15 to almost 50

percent of the Klebsiella pneumoniae problem of patients with blood infections were

resistant to the drugs. The figures were highest in Greece and Italy." (taz, 18.11.11)

The first criterion for determining a statement is the presence of an actor.

A statement can only be coded as a statement if it can be attributed to an identifiable actor (person, organization or identifiable community such as "experts").

A statement may only be attributed to a single actor. As soon as a new actor has their say, the

selection is to be coded as the next statement.

First and foremost, a statement is recognized by direct or indirect speech (paraphrase and

paraphrase), but also by attributing opinions, positions and actions:

Example: "The EU Commission has now presented an action plan to solve the problem.

In it, it proposes to the member states and the EU Parliament, for example, to

fight against infections in the healthcare sector." (taz, 18.11.11)

The second criterion for determining a statement is its thematic reference.

Only statements that deal with one of our science-related risk issues are coded as statements.

A statement may only have a single specific thematic reference. This means that the

same actor, who is mentioned several times in an article, can make several statements as long as they have different thematic references.

*3.2 Type of Statement*

Complex statements can be attributed to several categories at the same time. I.e. a statement can be simultaneously, action-related, substantiating and interpretative. In this case, all three types of statement are coded.

*3.2.1 Action-related statement*

Dichotomous variable. These are statements that are action-related in the broader sense. These can be calls for political intervention in the field of research, but they can also be statements directed at research institutions such as the DFG, for example to intensify research funding or expand it to include individual aspects. But it can also be a request to wash your hands, get vaccinated and the like. Finally, statements that refer to the choice of a specific research strategy should also be considered action-related statements.

Action-related statements answer the question: What should/can or should not/cannot be done to address the problem of the issue at hand (e.g. AMR/Ebola/pandemic flu and so on)?

1 = Yes

0 = No (continue with 3.2.2)

*3.2.1.1 Type of action-related statement*

We distinguish between three types of action-related statements:

*1 Political strategy*

These statements refer to political strategies and initiatives that aim to address a problem in connection with the respective issue at a political level. This category includes, for example, an EU initiative to monitor antibiotic prescribing or a supranational early warning and data collection system for Ebola.

Example: "Now fifteen representatives of scientific organizations in human and veterinary medicine from Germany have passed a resolution calling on the German government to ensure a Europe-wide ban on all antibiotics as feed additives." (SZ, 19.09.96)

A political strategic action is always determined by its "from top to bottom" direction. This means that the political initiative mentioned in the statement is or

can only be initiated and realized by political bodies (national, international or supranational).

*2 Collective effectiveness*

Statements in this category address various options for action on a collective or social level, however without being linked to any specific political program.

Examples: "Doctors must avoid unnecessary antibiotic prescribing", "The pharmaceutical industry must focus on the development of new antibiotics", "...we urgently need new antibiotics. In the past 30 years only two new classes of antibiotics have been discovered. It is a great challenge to reactivate the pharmaceutical industry's pipeline for the development of new antibiotics." (Die Welt, 06.02.12)

*3 Personal effectiveness*

These statements refer to the actions that individuals can take, such as "You have to wash the meat thoroughly from cooking."

NOTE: Sometimes it can be difficult to distinguish between collective and personal effectiveness. You have to pay attention to what is the target group. For example, it can be stated that the individual person should wash their hands regularly. In this case, it is an action with personal effectiveness. On the other hand, the text may state that all doctors and other hospital staff should wash their hands regularly. In this case, it is classified as an act with collective efficacy. In the same way, an action-related statement with personal effectiveness could receive a recommendation for individuals to wear a mask against influenza when they leave the house. On the other hand, an action-related statement with collective efficacy would advise all salespeople in large retail centers to wear masks. If general practitioners not to vaccinate patients because of side effects, in this case it is about collective efficacy. At the same time, if the federal government approves the legal basis "for the mass vaccination of up to 35 million German citizens against swine flu" it will be coded as a politically strategic action-related statement.

*3.2.2 Substantiating statement*

Dichotomous variable. These are statements that have a clear reference to the objectively

recognizable world, i.e. statements that can be true or false because they contain an element of fact. This is the case, for example, when prevalence rates are given, generally concrete figures, even if they are estimates, such as "the risk is increasing" (these can also be true or false, even if this cannot be decided at the moment). The references to the findings,

disease symptoms, development mechanisms of resistance/mutation of viruses and similar elements of objective knowledge that can be traced back to research are all to be regarded as substantiating statements.

A substantiating statement answers the questions: What exactly do we know? What is the object of our knowledge? How did this knowledge come about?

Example: "According to the Chilean agricultural authority SAG, the swine flu pathogen has

agricultural authority SAG, the swine flu pathogen has also been detected in birds for the first time." (SZ, 21.08.09)

1 = Yes

0 = No (continue at 3.2.3)

*3.2.2.1 Precision of the substantiating information*

This variable measures how precise the substantiating information is. A distinction is made here between three levels of precision. Qualitative data assesses the risk in a non-numerical way, e.g. through phrases such as "the risk is increasing", "the germs are becoming increasingly more dangerous" etc. Numerical data describes risk in absolute numbers, e.g. "during the outbreak, 7 people were infected with resistant germs", "in Germany there are 10,000 infected people per year", "5,000 hospitals"). Numerator-denominator data are highly precise data that always occur with a denominator: "2% of the population", "1 in 5

are infected"

1 = Qualitative data

2 = Numerator data

3 = Numerator-denominator data

*3.2.3 Risk interpretations*

These are statements that clearly refer to the risks associated with the issue at hand. This can be identified by words such as risk, danger, hazard and other semantically related terms and constructions. It also includes the representations of risk by e.g. mentioning infected persons, diseased persons and fatalities as well as other statistical information that indicates a potential risk situation.

Example: "Around 3000 people die from hospital germs in Germany every year"

Example: "Only 5% of hospitals have a hygienist"

Example: "A large proportion of the meat was contaminated with resistant germs"

Example: "RKI expects several thousand flu infections this year"

ATTENTION: It is very likely that the risk-related statements can also be used as

to be coded as substantiating statements.

1 = Yes

0 = No

*3.2.3.1* *Tenor of the risk interpretation*

We distinguish between an alarming and a non-alarming tenor of the risk-related statement.

The tenor is alarming if the statement is aimed at concrete or potential damage. In order to have an alarming tenor, it is not necessary that any dramatizations are included, these at best reinforce the tenor.

Example: "HIP and knee operations are becoming "life-threatening" because of the rise of antibiotic resistance, a report by public health officials warns today. (...)" (The Daily Telegraph, 23.10.2018)

Statements that list damages or those affected in a relatively factual manner, such as the next example article, also have an alarming tone, because in this case the statements are directed exclusively at specific or potential damage.

Example: “The HSE has changed the national treatment guidelines for gonorrhoea because testing has revealed that the sexually transmitted disease is becoming resistant to antibiotics in Ireland. The incidence of gonorrhoea has grown sharply in recent years, rising by 16% in 2017 and 7% in 2018 to 2,407 diagnoses. Scientists at the National Gonococcal Reference Laboratory in St James's Hospital have warned the disease could become untreatable if antibiotics fail. Until late last year, gonorrhoea was typically treated with a dual therapy of two antibiotics, azithromycin and ceftriaxone. However, the bacteria that causes gonorrhoea is becoming resistant to both of these. The St James's Hospital scientists reported that 13% of samples it tested between 2010 and 2017 were resistant to azithromycin, with the rate of resistance above the European average since 2014. A case of ceftriaxoneresistant gonorrhoea was identified in Ireland last year. As a result, gonorrhoea cases will now be treated with ceftriaxone alone, at double the dosage previously prescribed, to ensure it is effective on less susceptible strains.” (The Sunday Times, 10.03.2019)

1 = alarming

0 = not alarming

# 4 Reliability values

**Table S1. Reliability values**

| **Variable** | **Krippendorff’s alpha** |
| --- | --- |
| Specification of the actor | 0.88 |
| Name of the actor | 0.97 |
| Institutional affiliation of the actor | 0.87 |
| National affiliation of the actor | 0.89 |
| Actor’s affiliation to a societal area | 0.78 |
| Action-related statement | 0.79 |
| Substantiating statement | 0.74 |
| Risk interpretation | 0.73 |

# 5 Sampling procedure

## 5.1 Dioxin

Up to 160 articles per source were randomly selected out of all articles published between January 1999 and December 2019 in Die Welt, Süddeutsche Zeitung, Der Spiegel and dpa. The German keyword used for the search was: Dioxin*. Only articles in which dioxin was the main topic were included in the actor analysis.

## 5.2 Glyphosate

Up to 320 articles per source were randomly selected out of all articles published between January 2015 and December 2019 in Die Welt, Süddeutsche Zeitung, Der Spiegel and dpa. The German keyword used for the search were: glyphosat* OR herbicid*. Only articles in which glyphosate the main topic were included in the actor analysis.

## 5.3 Nitrogen oxids

Up to 160 articles per source were randomly selected out of all articles published between October 2011 and June 2019 in Die Welt, Süddeutsche Zeitung, Der Spiegel and dpa. The German keyword used for the search were: ((stickoxid*) OR (stickstoffoxid*) OR (stickstoffmonoxid*) OR (stickstoffdiox*) OR (NOx)). Only articles in which nitrogen oxides were the main topic were included in the actor analysis.

## 5.4 Marijuana

Up to 160 articles per source were randomly selected out of all articles published between January 2012 and May 2019 in Die Welt, Süddeutsche Zeitung, Der Spiegel and dpa. The German keyword used for the search were: (Marihuana* OR Cannabi*) AND Legalisier*. Only articles in which marijuana was the main topic were included in the actor analysis.

## 5.5 Ebola

15 % of all articles identified with the search string and published between January 1995 and December 2015 by Die Welt, Süddeutsche Zeitung, Der Spiegel and dpa were randomly selected. The German keyword used for the search was: Ebola*. Only articles in which Ebola was the main topic were included in the actor analysis.

## 5.6 Flu pandemics

15 % of all articles identified with the search string and published between January 1995 and December 2015 by Die Welt, Süddeutsche Zeitung, Der Spiegel and dpa were randomly selected. The German keyword used for the search were: schweinegrippe oder (pandemie AND grippe). Only articles in which influenza was the main topic were included in the actor analysis.

## 5.7 AMR

All articles published in Die Welt, Süddeutsche Zeitung, Der Spiegel and dpa from every fourth year since 1997 (1997, 2001, 2005, 2009, 2013) were recorded. The German keyword used for the search were: antibio* AND resist*. Only articles in which antimicrobial resistance was the main topic were included in the actor analysis.

## 5.8 COVID-19

We first identified all relevant articles published between January and December 2020 in Die Welt, Süddeutsche Zeitung, Der Spiegel and dpa with our search string. The German keyword used for the search were: COVID-19: Corona*, Covid*, Corvid*, nCov*, n-Cov*, SARS*, Wuhan und Lunge*, Wuhan und Krankheit, China und Krankheit, China und Lunge*. The population was then split into four groups, which are not relevant for the content of the present paper (since we do not discuss or analyze potential differences between these phases) but which were needed in the context of other analyses:

- The preliminary phase until a pandemic was declared by the WHO on March 11th 2020 (Week 11). In this phase, 8 percent of the dpa articles and 15 percent of all other articles were randomly selected.

- This determination was followed by successive decisions by the federal and state governments to restrict social contact. The number of COVID-19 articles increased rapidly during this peak phase. It ends on April 15th, 2020 (Week 15). In this phase, 4 percent of the dpa articles and 10 percent of all other articles were randomly selected.

- In the 16th calendar week, the decisions to relax the contact bans were announced by the federal government in Germany. In this phase, 2 percent of the dpa articles and 5 percent of all other articles were randomly selected.

-The fourth and last phase starts with calendar week 25 and lasted until the end of 2020. In this phase, the media coverage was not characterized by any noticeable peaks or lows, which is why it was not further subdivided. In this phase, too, around 2 percent of the dpa articles and 11 percent of all other articles were randomly selected.

Only articles in which COVID-19 was the main topic were included in the actor analysis.

# 6 Formation of the control sample

Table S2 provides details on the composition of the control sample.

**Table S2. Number of contributing experts from publications with at least one German affiliation, from which a random sample of 300 experts each was selected**

| **Issue** | **N Experts** | **Searchstring** |
| --- | --- | --- |
| AMR | 6669 | (antibiotic* OR antimicrobial*) resist* |
| Corona | 10,783 | virology OR (epidemiology and infectious diseases) |
| NOX | 6621 | nitrogen dioxide OR "nitrogen oxide" OR NOX |
| Glyphosat | 534 | Glyphosate |
| Dioxin | 5875 | (dioxin* OR PCB OR PCDD OR PCDF OR furan* OR polychlorinated byphenil* OR polychlorinierte* Byphenil* OR TCDD OR TCDF OR PBDD OR PBDF OR PFDD OR PFDF) |
| Pandemic flu | 3029 | Influenza |
| Ebola | 382 | (ebola* OR filovir*) |
| Marihuana | 3108 | Cannabi* OR Marihuana* OR Marijuana* OR Dronabinol* |

# 7 Automated gender coding via Namsor

The gender of the scientific actors was automatically coded using the platform Namsor (https://namsor.app/). In addition to a binary gender classification for a name, Namsor also returns a certainty for this classification in form of a value between 50 and 100 percent. We tested the accuracy of the automated classification by having three coders manually categorize a total of 1728 personal names. We then had the same names encoded automatically via Namsor. In all cases for which the certainty of the procedure was above 90 percent, the agreement between manual and automated coding was .99 according to the Holsti coefficient respectively .977 according to Krippendorff's alpha. Thus, in such cases we are confident that the automated classification is accurate. All cases in which certainty was below 90 percent were coded manually (213 cases), as were 100 cases for which only initials were available. Here the procedure was as follows: Firstly, an attempt was made to make a classification based on the name and personal knowledge of whether a name is more commonly used for male or female persons. If coders were unsure, they secondly searched for the scientist’s name using *Google* and looked at up to three hits to arrive at a classification.
